# Fingolimod Alters Tissue Distribution and Cytokine Production of Human and Murine Innate Lymphoid Cells

**DOI:** 10.3389/fimmu.2019.00217

**Published:** 2019-02-13

**Authors:** Ahmet Eken, Mehmet Fatih Yetkin, Alperen Vural, Fatma Zehra Okus, Serife Erdem, Zehra Busra Azizoglu, Yesim Haliloglu, Mustafa Cakir, Enes Mehmet Turkoglu, Omer Kilic, Irfan Kara, Hamiyet Dönmez Altuntaş, Mohamed Oukka, Mehmet Serdar Kutuk, Meral Mirza, Halit Canatan

**Affiliations:** ^1^Erciyes University School of Medicine, Department of Medical Biology, Kayseri, Turkey; ^2^Betül-Ziya Eren Genome and Stem Cell Center (GENKOK), Kayseri, Turkey; ^3^Department of Neurology, Erciyes University School of Medicine, Kayseri, Turkey; ^4^Department of Ear Nose and Throat, Erciyes University School of Medicine, Kayseri, Turkey; ^5^Department of Immunology, University of Washington, Seattle, WA, United States; ^6^Department of Obstetrics and Gynecology, Erciyes University School of Medicine, Kayseri, Turkey

**Keywords:** S1PR1, ILC3, ILC1, Fingolimod, FTY720, SEW2871, multiple sclerosis

## Abstract

Sphingosine-1 phosphate receptor 1 (S1PR1) is expressed by lymphocytes and regulates their egress from secondary lymphoid organs. Innate lymphoid cell (ILC) family has been expanded with the discovery of group 1, 2 and 3 ILCs, namely ILC1, ILC2 and ILC3. ILC3 and ILC1 have remarkable similarity to CD4+ helper T cell lineage members Th17 and Th1, respectively, which are important in the pathology of multiple sclerosis (MS). Whether human ILC subsets express S1PR1 or respond to its ligands have not been studied. In this study, we used peripheral blood/cord blood and tonsil lymphocytes as a source of human ILCs. We show that human ILCs express S1PR1 mRNA and protein and migrate toward S1P receptor ligands. Comparison of peripheral blood ILC numbers between fingolimod-receiving and treatment-free MS patients revealed that, *in vivo*, ILCs respond to fingolimod, an S1PR1 agonist, resulting in ILC-penia in circulation. Similarly, murine ILCs responded to fingolimod by exiting blood and accumulating in the secondary lymph nodes. Importantly, *ex vivo* exposure of ILC3 and ILC1 to fingolimod or SEW2871, another S1PR1 antagonist, reduced production of ILC3- and ILC1- associated cytokines GM-CSF, IL-22, IL-17, and IFN-γ, respectively. Surprisingly, despite reduced number of lamina propria-resident ILC3s in the long-term fingolimod-treated mice, ILC3-associated IL-22, IL-17A, GM-CSF and antimicrobial peptides were high in the gut compared to controls, suggesting that its long term use may not compromise mucosal barrier function. To our knowledge, this is the first study to investigate the impact of fingolimod on human ILC subsets *in vivo* and *ex vivo*, and provides insight into the impact of long term fingolimod use on ILC populations.

## Introduction

S1P receptor 1 (S1PR1) is a G-protein coupled receptor expressed by endothelial cells and lymphocytes. Upon binding to its natural ligand S1P, S1PR1 activates various signaling cascades, including Ras–Erk, PI3K–Akt, and the small G proteins Rac and Rho (1, 2). S1PR1 plays a critical role in the egress of T and B cells out of thymus and lymphoid organs (3–5). Egress regulation is achieved by a gradient of S1P across lymphoid tissues and blood or lymph, which is created by tight regulation of S1P production by kinases SPHK1 and SPHK2 and its reversible or irreversible degradation by S1P lyase or phosphatases, respectively (2, 6–8). Multiple S1PR1 agonists/antagonists have been discovered. Fingolimod (FTY720 or Gilenya™) is a structural analog of sphingosine-1 that blocks lymphocyte egress into blood or lymph by inducing S1PR1 internalization (9). Fingolimod was approved by FDA in 2010 as the first oral disease modifying pill for the treatment of MS. FTY720, however, is not very specific and can bind S1PR1, S1PR3, S1PR4, and S1PR5 (9). Thus, adverse effects including bradycardia have been reported (10). Moreover, although approved for the treatment of MS (11), in some patients, cessation or initiation of fingolimod therapy resulted in exacerbation of MS and formation of tumefactive lesions in the brain (12–16). Other S1PR1 agonists such as ponesimod, siponimod, and ozanimod are also in clinical trials recruiting MS patients (17–19). Ozanimod and siponimod are more specific than fingolimod and bind S1PR1 and S1PR5. S1PR1 antagonists/agonists are also tested for other conditions besides MS, including psoriasis, graft vs. host disease (GVHD) and inflammatory bowel diseases (IBD) (17–19). Therefore, the potential for the broader use of S1PR1 modulators for the treatment of a host of autoimmune conditions underlines the need for a better understanding of S1PR1 functions in various cell types in the body.

In this regard, the role of S1PR5 in murine natural killer (NK) cells has been studied (20). A report demonstrated that the tissue distribution of NK cells was regulated by S1PR5, thus S1PR5-deficient NK cells are enriched in the lymph nodes (LNs) and bone marrow (BM) and are reduced in circulation. Human NK cells also have recently been shown to use S1PR5 (21). Innate lymphoid cell family (ILC) has recently been expanded with the discovery of helper-T-cell-like innate lymphoid cell subsets ILC1, ILC2, and ILC3s (22–29). ILC3s emerged as the innate counterpart of Th17 cells. As such, ILC3s have been shown to play an essential role at the mucosal surfaces by establishing a de-colonized zone between the microbial community and the epithelial cells through IL-22 cytokine they produce (30, 31). In addition, ILC3s were shown to be crucial in fighting infections in murine models (32). More importantly, ILC3 involvement in the chronic inflammatory diseases such as Crohn's and colitis have been established in murine models as either pathogenic or protective agents in our and others' works (33–41). Increased ILC3 cellular presence or activity have been reported in the affected tissues or circulation in several other autoimmune disease patients from rheumatoid arthritis, psoriasis to MS warranting further study of these cells in human pathologies (42, 43). Although a population of ILC3s express NKp46 in mice and NKp44 in humans, it is completely unknown how S1P receptors regulate human or murine ILC3 biology or how drugs against S1P receptors would impact a critical mucosal innate cell population.

In the present study, we aimed to understand the role S1P receptors play in ILC biology, tissue distribution and function using tonsil or cord blood derived ILCs, fingolimod receiving MS patients and murine models. We show that S1PR1 may regulate human ILC1 and ILC3 chemotaxis and that S1PR1 ligands fingolimod and SEW2871 have immunomodulatory effects on human ILC3s. We also show that although long term fingolimod treatment of mice reduces intestinal ILC3 numbers, it does not reduce IL-17A, IL-22 or antimicrobial peptides, sparing the barrier immunity intact.

## Materials and Methods

### Human Samples

Peripheral blood samples were taken from patients at Erciyes University School of Medicine, Department of Neurology. Informed consent was obtained from donors after the nature and possible consequences of the studies were explained. Revised McDonald Diagnostic Criteria for MS (Diagnosis of multiple sclerosis: 2017 revisions of the McDonald criteria) was used for diagnosis of RRMS (44). All subjects in the MS patient/no treatment cohort were off immunomodulatory and immunosuppressive drugs at the time of study and for about 3 months before the blood samples were taken. Control subjects lack any known autoimmune condition or a family history of autoimmunity. Tonsils were taken after they were being discarded by Erciyes University Hospital Surgery Department. Cord Blood samples were taken from discarded cord following labor at Erciyes University Gevher Nesibe Hospital. The research protocols were approved by the Ethics Committee at Erciyes University. All methods for human studies involving human samples were performed in accordance with the relevant guidelines and regulations.

### Mice

The research protocols involving mice were approved by the Animal Ethics Committee at Erciyes University. All methods for mice studies were performed in accordance with the relevant guidelines and regulations. C57BL/6 or IL-23RGFP reporter mice were housed under specific pathogen free conditions. 0.5 mg/kg fingolimod was administered daily intraperitoneally for 15-to 30 days. As control, PBS was given. SEW2871 (10006440 256414-75-2) and FTY720-P (10008639) were purchased from Cayman. For gavage feeding, Gilenya (Novartis) 0.5 mg tablets were reconstituted in Phosphate-Buffered Saline (PBS) and administered daily at 0.5 mg/kg dose. One hundred microgram (in 100 μl PBS) anti-CD40 (FGK4.5, Bioxcell) was injected intraperitoneally 2-days prior to sacrificing mice.

### Isolation, Staining, and Culture of Cells

Peripheral blood mononuclear cells (PBMCs) were isolated from blood via Ficoll-Paque Plus (GE17-1440-03) based on the manufacturer's instructions. Mice lymphocytes from lymph nodes, spleen or intestines were isolated as previously described (45). For intestinal lamina propria preps, 2–3 cm piece from ileum was processed for lamina propria lymphocyte isolation. Single cell suspension was stained for the relevant antibodies after blocking 5 min with Human TruStainFcX (BioLegend, San Diego, CA, USA) in Staining Buffer (2% FBS in PBS) with proper dilution. The cells were. gated and sorted via FACSAria III (BD Biosciences) according to the protocol by Mjösberg and Spits (46).

Human ILC3s were cultured in the following cocktail of cytokines, IL-2, IL-7, IL-23, IL-1β, 20 ng/ml each (47). ILC1 cells were cultured in the cocktail containing cytokines, IL-2, IL-7, IL-12, 20 ng/ml each.

### Antibodies and Reagents

Alexa Fluor® 700 anti-human CD3, (clone: HIT3a), FITC-anti human TCRαβ (clone: IP26), FITC-anti-human TCRγδ (clone: B1), APC/Cy7 Anti-Human CD127 (I-7Ra), (clone: A019D5), PE anti-human CD161, (clone: HP-3G10), Brilliant Violet 421TM anti-human CD117 (c-kit), (clone 104D2), PE/Cy7 anti-human CD294 (CRTH2), (clone: BM16), APC anti-human CD336 (NKp44), (clone: 325110), Alexa Fluor® 488 anti-human CD19, (clone: HIB19), FITC anti-human CD94, (clone:DX22), FITC anti-human CD1a, (clone: HI149), FITC anti-human CD11c, (clone: 3.9), FITC anti-human CD123, (clone: 6H6), anti-human CD303 (BDCA-2), (clone:201A), FITC anti-human CD14, (clone: 63D3), FITC anti-human FcεRIα, (clone: NP4D6), FITC anti-human CD34,(clone: 561), APC/Cy7 anti-human IFN-γ, (clone: 4S.B3) all from BioLegend. Anti-Human CD363 (S1PR1) eFluor® 660, (clone: SW4GYPP, ThermoFisher), Mouse IgG1 K Isotype Control eFluor® 660, (clone: P3.6.2.8.1, ThermoFisher). Anti-mouse CD3) APC, (clone: 17A2), anti-mouse NK1.1 Alexa Fluor® 647, (clone: PK136), anti-mouse B220 APC/Cy7 or FITC, (clone:RA3-6B2), anti-mouse CD45-Percp Cy5.5, (clone:30-F11), anti-mouse CD90.2 PE Cy7, (clone:30-H12), anti-mouse CD11b APC, (clone:M1/70), anti-mouse GATA3 PE (Clone: 16E10A23), anti-mouse Rorγt (Clone: 2F7-2).

### Real-Time qPCR

Sorted or cultured ILC subsets were spun and lysed with lysis buffer from RNeasy kit (Qiagen, Hilden, Germany), in most studies 3–5 samples were pooled. RNA was extracted. cDNA was synthesized using iScript cDNA synthesis Kit (Bio-Rad, Hercules, CA, USA). Light Cycler® 480 Instrument and SYBR Green method was used to detect PCR products. Relative gene expression levels were calculated by ΔΔCT method. Expression was normalized over 18S ribosomal RNA message. Primer sequences are given in Figure S6.

### Chemotaxis Assay

Briefly, sorted ILC subsets were rested in serum free media for 4 h at 37°C (5% CO_2_). Then 1 × 10^4^ to 2 × 10^4^ cells per insert (depending on the availability of sorted cell number) loaded into the polycarbonate inserts (Corning) with 5 μm pores in 300 μl of serum free medium. In the bottom wells, 500 nM ligand containing medium was added. As control, serum free media, used. After 4 h of culture at 37°C, number of cells migrating to the wells was quantified by Flow cytometry and charted as percent migrated cell vs. treatment. For pretreatment experiments, ILCs were cultured for 2 h in the presence of serum free media (RPMI 1640, Gibco), or media with 1 μm FTY720, or SEW2871, after they are washed, the cells were seeded in the inserts. At the bottom, 1 μm FTY720 was added. Cells migrating to bottom wells were counted by Flow cytometry, SPHERO™ AccuCount Particles.

### Enzyme-Linked Immunosorbent Assay (ELISA)

Sorted human ILCs were cultured in 96-well plates at 37°C, 5% CO2 incubator for 2–3 days in medium supplemented with charcoal stripped FBS and ILC polarizing cytokines as described above. The supernatants were collected for ELISA. For whole colon/small intestine cultures, 1 cm piece of tissue from the ileum, or proximal colon was cut from fingolimod or vehicle-treated mice. The tissues were cleaned under sterile conditions as previously described (37) and cultured in 500 μl complete media supplemented with anti-anti at 37°C, 5% CO_2_ incubator for 2 days in 24-well plates. Supernatants were collected for ELISA. Collected supernatants from human cells or mouse intestinal tissues were used to run ELISA for human or murine IL-22 (BioLegend #434505 and #436305), IL-17A (BioLegend #433915 and #432505), and GM-CSF (BioLegend #432005 and #432205), respectively. Manufacturer's protocol was followed.

### Statistical Analyses

Two tailed, Unpaired Student's *t*-test and GraphPad Prism 6 was used for significance analyses. *P* < 0.05 is accepted as statistically significant.

## Results

### Human ILC1 and ILC3 Express S1PR1 and Respond to Its Ligands

**Figure 1:**
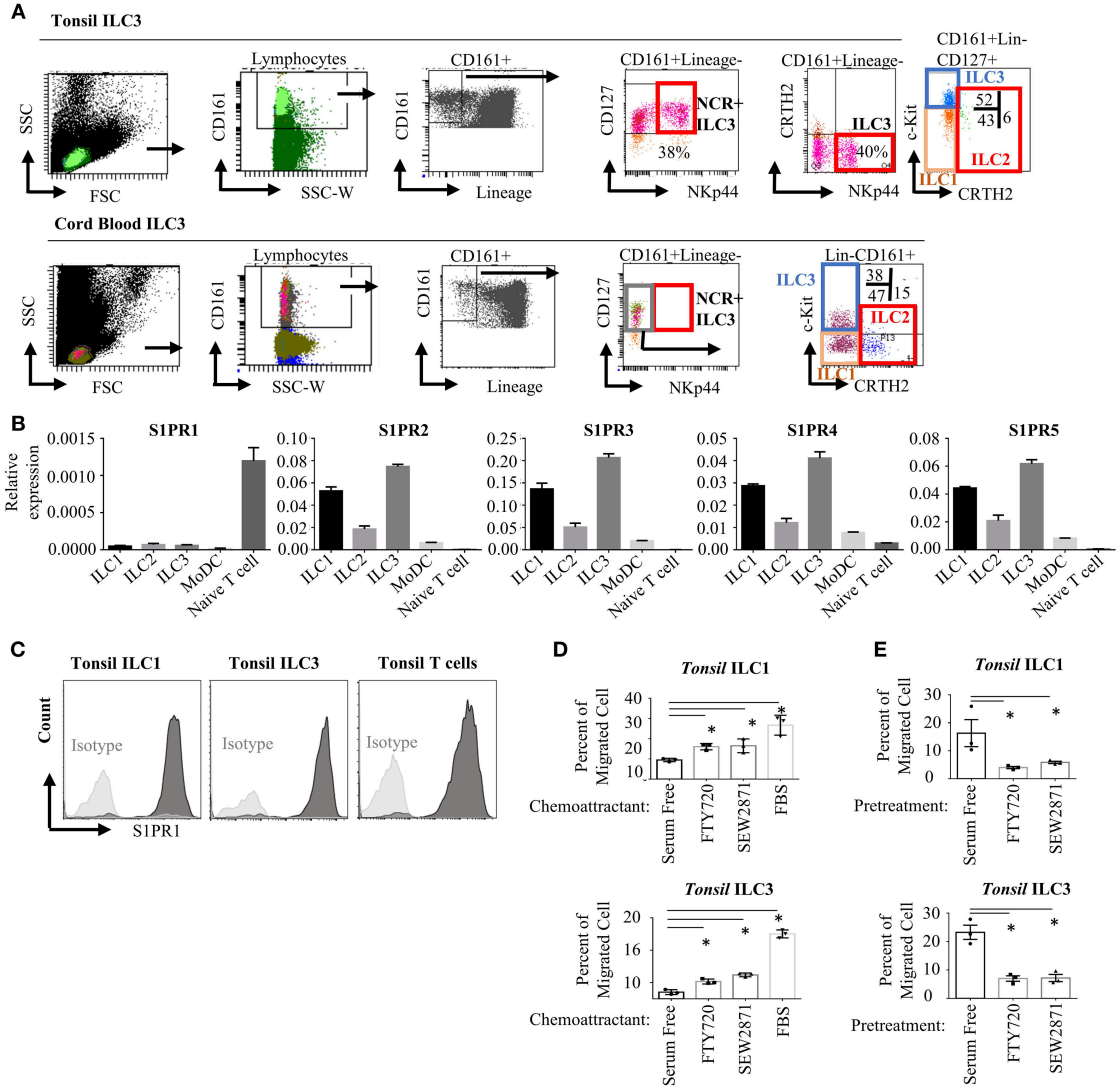
Human ILC subsets express S1P receptors and migrate toward S1P analogs *in vitro*. **(A)** Gating strategy for human ILC subset isolation from tonsil and cord blood. **(B)** Sorted ILC subsets were used to determine relative gene expression of S1P receptors (S1PR1, S1PR2, S1PR3, S1PR4, S1PR5) via real time qPCR. **(C)** Human tonsil ILC1 and ILC3 were stained for S1PR1 to determine protein expression, a representative histogram flow plot with isotype control. **(D)** Migration of ILC1 and ILC3 cultured in serum free media toward FTY720 and SEW2871 gradient. Percentage of cells migrating into lower chamber of a trans-well plate quantified. **(E)** Migration of ILC1 and ILC3 pretreated with serum free media, FTY720 or SEW2871 for 2 h toward FTY720. Percentage of cells migrating was charted. *p < 0.05. Experiments in **(B–E)** were performed three separate times.

To assess the expression and functionality of S1P receptors in human ILC subsets, we first analyzed the RNA-Seq and microarray datasets available at GEO database for murine intestinal ILC3s and Th17 cells (48) and human tonsil ILC3 (49) and NK cells. Previously S1PR5 was shown to be expressed more abundantly than other S1P receptors and to regulate NK cell lymphoid tissue egress (20, 21). We observed very low S1PR5 message by both murine and human ILC3s compared with other S1P receptors (Figure S1A). We then, sort purified ILCs from human tonsils and cord blood since these two organs have been documented to contain relatively enriched number of ILCs. ILC gating was done based on Mjösberg and Spits (46). Lineage negative (TCRαβ-, TCRγδ-, CD34-, CD123-, CD94-, CD14-, BDCA2-, FcεRIα-, CD1a-, CD11c-, CD19-, B220-) CD3-CD161+CD127+cKit+ cells were sorted as ILC3, Lineage-CD3-CD161+CD127+cKit- cells were sorted as ILC1 and Lineage-CD3-CD161+CD127+CRTH2+ cells sorted as ILC2 (Figure 1A). As documented, human tonsils contained around 40 % NKp44+ ILC3s, whereas cord blood contained mostly NKP44- ILC3 fraction (50). Cord blood or peripheral blood ILC3 gate has been recently shown to contain precursors for all ILCs and named as precursor ILCs (pILCs). This should be kept in mind when blood ILC3s were discussed in the remainder of the paper. Sorted ILC1, ILC2, and ILC3 from tonsils expressed S1PR2-S1PR5 receptors mRNA at higher levels than T and monocyte derived dendritic cells (moDC). In contrast, S1PR1 message levels were significantly higher in T cells compared with ILCs and moDC. S1PR1 gene expression in human ILCs were comparable to that of moDCs (Figure 1B). Compared to other S1P receptors S1PR1 expression by human ILCs were low. We also examined the expression of S1P receptors by real time qPCR in the murine intestinal ILC3 and ILC2/ILC1s. Similar to humans, S1PR1 was expressed at lower levels than other S1P receptors, however murine ILC3 and ILC2/ILC1s expressed comparable S1PR1 to intestinal T cells (Figure S1B). Flow cytometric assessment of S1PR1 expression revealed high level S1PR1 protein expression by human ILC1 and ILC3s on the cell surface (Figure 1C and Figure S1E). We next tested the functionality of the S1P receptors in human ILC1 and ILC3 by a chemotaxis assay. To this end, we used two analogs of S1P, FTY720-P (fingolimod) and SEW2871. FTY720 binds four of five S1P receptors (but not S1PR2), SEW2871 however is more specific and binds exclusively S1PR1. Both ILC1s and ILC3s were able to migrate toward high gradients of both FTY720 and SEW2871, or fetal bovine serum (which contains high levels of S1P) compared with serum free media suggesting that S1PR1 may regulate human ILC1 and ILC3 chemotaxis (Figure 1D). Furthermore, to assess which S1P receptors used by ILCs to migrate, we pre-treated ILCs with serum free media, FTY720 (1 μM) or SEW2871 (1 μM) for 2 h. The latter two treatments are expected to cause internalization of four S1P receptors (S1PR1, S1PR3, S1PR4, and S1PR5) or S1PR1 alone, respectively, rendering ILCs unresponsive to S1P. Such pre-treatment of ILC1 and ILC3 with SEW2871 blocked migration almost as efficiently as FTY720 pretreatment, suggesting that S1PR1 plays a major role in the migration of human ILC1s and ILC3s (Figure 1E). Additionally, treatment of ILC1 with pertussis toxin, a G-protein coupled receptor inhibitor, blocked cell migration toward fingolimod suggesting that S1PR1 may regulate human ILC migration (Figure S1C). Although pretreatment of ILCs with FTY720 or SEW2871 blocked their migration toward FTY720 *ex vivo*, the cells were able to migrate toward serum even after pretreatment FTY720 or SEW2871 suggesting other ligands and receptors may compensate their migration *ex vivo* (Figure S1D) (51). These data collectively show that human ILC1s and ILC3s express S1P receptors and thus respond to ligands *ex vivo*, and that S1PR1 plays an important role in human ILC1 and ILC3 migration. Unfortunately, we could not include ILC2 in our chemotaxis assays due low yield of cell number in the sorts.

### Fingolimod Receiving MS Patients Have ILC-Penia

**Figure 2:**
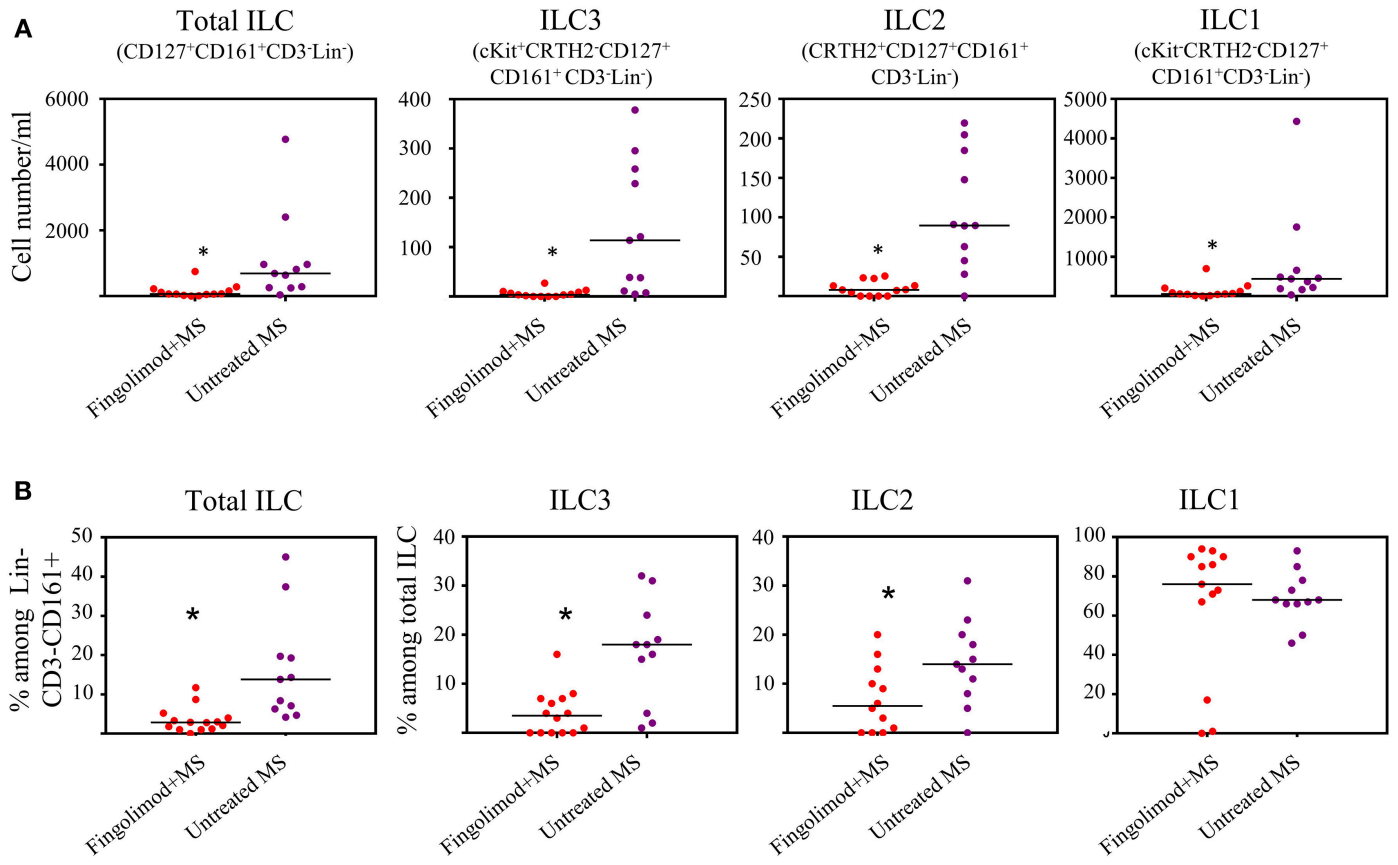
Fingolimod causes ILC-penia in human peripheral blood. **(A)** absolute number of total or subsets of ILCs in the peripheral blood of MS patients treated with fingolimod (*n* = 14) or untreated control MS patients (*n* = 11) per 10 ml peripheral blood. Total ILCs were gated as CD127^+^CD161^+^CD3^−^Lin^−^ cells, ILC3s as cKit^+^CRTH2^−^CD127^+^ CD161^+^ CD3^−^Lin^−^, ILC2s as CRTH2^+^CD127^+^CD161^+^ CD3^−^Lin^−^, and ILC1s as cKit^−^CRTH2^−^CD127^+^ CD161^+^CD3^−^Lin^−^. **(B)** percentages of ILCs in “A”. *Indicates *p* < 0.05.

Next, we explored whether human ILCs respond to S1P agonists *in vivo*. To this end we recruited 14 patients with relapsing remitting MS (RRMS) who are under fingolimod therapy and compared blood ILC subset numbers to that of RRMS patients who were not receiving fingolimod (n = 11). Age and sex information for the patients were provided in Table S1. Gating strategy for human peripheral blood ILCs is shown in Figure S2A. We observed dramatic reductions in the absolute number of total ILCs, and all subsets of ILCs in the peripheral blood of MS patients who are under fingolimod therapy compared to control MS patients not on fingolimod (Figures 2A,B). The percentages of total ILCs among CD161^+^CD3^−^Lin^−^ cells, and of ILC1s and ILC2s among CD127^+^CD161^+^CD3^−^Lin^−^ total ILCs were also reduced. These data collectively indicate that human ILCs respond to S1P receptor agonists and suggests that fingolimod sequesters peripheral blood human ILCs in LNs.

### Fingolimod Alters ILC Distribution Across Lymphoid Organs and Gut in Mice

**Figure 3:**
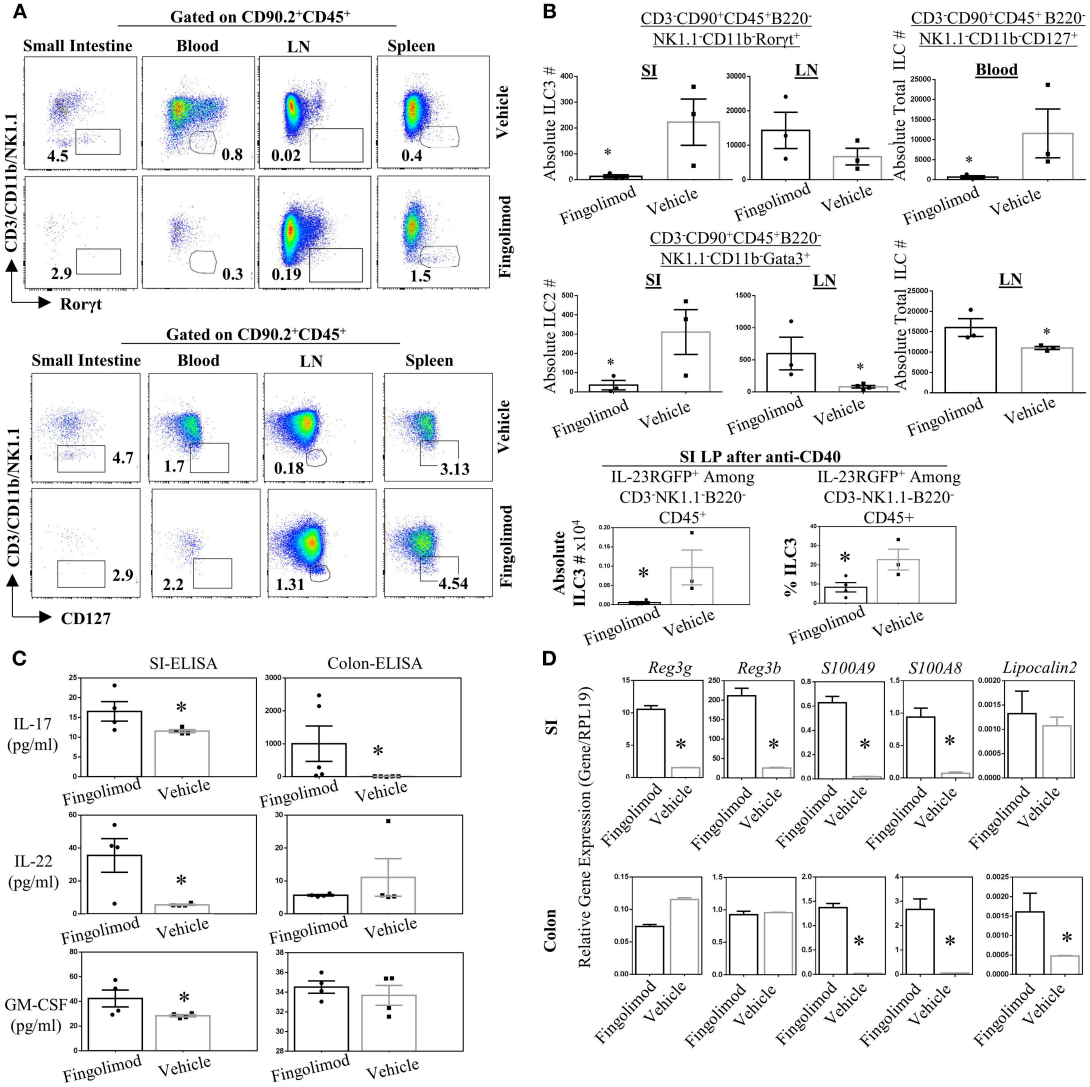
Fingolimod causes ILC-penia, augments lymph node ILC numbers, and decreases small intestine lamina propria ILC3 numbers in mice but does not reduce antimicrobial peptide production. **(A)** A representative flow plot of the percentages of total ILCs in the small intestine lamina propria blood, inguinal lymph node (LN) and spleen of mice gavage-fed with fingolimod or vehicle for 30 days. The plots show live cells gated on CD90.2+CD45+B220- which then plotted as CD3/NK1.1/CD11b vs. Rorγt (top panel) or Gata3 (bottom panel). **(B)** Absolute number of Rorγt+ ILC3s (top panel), of Gata3+ ILC2s (middle panel) in the steady state mice after fingolimod of vehicle-gavage feeding for 30 days. Top panel, right, shows total ILCs (CD90.2-CD45+CD3-B220-CD11b-NK1.1-CD127+) in the blood of steady state mice following 30 days of fingolimod or vehicle treatment. The bottom most panel shows absolute number or percentages of IL-23RGFP+ ILC3s in the small intestine lamina propria 2-days after anti-CD40 injection following 30 days fingolimod or vehicle treatment. Three-to-four mice per group were used. Experiment was repeated 3 times. **(C)** 1 cm piece of ileum from fingolimod or vehicle-injected mice (for 30 days) were cultured 48 h and the supernatants were assessed with ELISA for the production of indicated ILC3-associated cytokines. Four to five of mice per group. **(D)** 1 cm piece of ileum or colon from fingolimod or vehicle-injected mice for 30 days was examined for gene expression of indicated antimicrobial peptides via real-time qPCR. *Indicates *p* < 0.05.

Recently Huang et al. reported that murine ILC2 tissue trafficking is regulated by S1P, especially the egress from intestinal tissue to lung during inflammation (52). Yet it is unclear if, in humans or murine models, long term fingolimod use alters ILC3 distribution across tissues, and consequently the associated cytokine production. Given the critical role of ILC3s in the establishment of barrier immunity, we quantified the number of ILC subsets in the lymphoid organs and gut of healthy B6 mice treated daily with fingolimod intraperitoneally for 30 days (Figure 3). Then the mice were sacrificed to harvest organs in a non-inflammatory setting. In some experiments, 2 days prior to terminating the experiment, we injected mice intraperitoneally with 100 μg of anti-CD40 to expand and boost the detection of gut ILC3s (37). anti-CD40 injection have been shown to promote IL-23 production by DCs and expand ILC3s. in the gut (37). Murine ILC detection was done based on our previous publication (45) and gate strategy for every organ is given in Figure S3. After B220-CD45+CD90.2+ cells were gated, CD3-CD11b-NK1.1- Rorγt+ cells were defined as ILC3s, and CD3-CD11b-NK1.1-Gata3+ cells defined as ILC2s. CD3-CD11b-NK1.1-CD127+ cells were accepted as total ILCs. We also used IL-23RGFP reporter mice for fingolimod injections/gavage feeding which marks ILC3s without the need for intracellular Rorγt+ staining (Figures S3B,C). In the blood, using either IL-23RGFP mice or Rorγt+ staining, detection of a specific classical ILC3 was not possible (Figure 3A, Figures S2B, S3A). Similarly, using Gata3 staining, detection of a specific ILC2 subset was also difficult (Figure S4A). For these reasons, we gated CD90.2+CD45+CD3-CD11b-NK1.1-CD127+ fraction to mark total blood ILCs or ILC precursors. This fraction of blood ILCs were significantly diminished following fingolimod injections (Figure 3B). Conversely, in the inguinal LN of mice injected with fingolimod we detected significantly higher absolute numbers of total ILCs (Figure 3B), suggesting that, similar to T and B cells, they rely on S1P receptors for egress from secondary lymphoid organs (Figures 3A,B). Moreover, in the lamina propria of small intestine and LN we could show significantly lower number of Rorγt+ILC3s and Gata3+ILC2s after 30 days of Fingolimod injections (Figures 3A,B and Figure S4A). This reduction in ILC3s in the small intestine could also be recapitulated after anti-CD40 injections (Figure 3C, bottom panel; Figure S4C). The changes in the frequency of ILCs in the blood, lymph nodes or small intestine lamina propria upon fingolimod treatment were similar to those of CD45+ lymphocytes, T and B cells, suggesting that fingolimod affects distribution of most lymphocytes in a similar way, Figures S4B,F. Importantly, fingolimod treated mice did not show any observable health issues or weakness during the 20–30 days of treatment.

Genetic deletion or antibody-mediated depletion of ILC3s from mice was previously associated with bacterial translocation across intestine, and into blood (53). Therefore, to further assess whether fingolimod-induced intestinal ILC3 depletion translates to any changes in the barrier immunity, we quantified the expression of various ILC3-associated cytokines (IL-17A and F, IL-22, GMCSF, IL-10) and antimicrobial proteins (Reg3γ, Reg3B, S100A9, S100A8) in the intestines of mice following fingolimod injections (Figures 3C,D). To our surprise, we detected high levels of anti-microbial Reg3γ, Reg3B, S100A9, S100A8 in the ileum of mice treated with fingolimod intraperitoneally compared with controls. In the colon, only S100A9, S100A8, and lipocalin 2 messages were highly upregulated. Intestinal *ex vivo* culture supernatants also revealed significantly higher IL-17A, IL-22, and GM-CSF protein production by the fingolimod treated group suggesting that despite the drop in the cell number, the intestinal tissue of mice which received intraperitoneal fingolimod is able produce antimicrobial peptides and IL-22 or IL-17A.

We next wondered whether route of fingolimod administration had an effect in our observations. Therefore, we gavage-fed the mice with fingolimod for 2-weeks and then following anti-CD40 treatment, quantified the number of ILCs in the gut, and examined gut and skin tissue for antimicrobial peptide and ILC3-associated cytokine production (Figures S4C–E). The results were almost identical to that of mice treated intraperitoneally with fingolimod. ILCs were greatly reduced in number in the colon and small intestine lamina propria in orally fingolimod-treated mice compared with controls (Figure S4C). More importantly, both in the gut and skin, antimicrobial peptides Reg3γ, Reg3B, and IL-22 cytokine gene expression were upregulated (Figures S4C,E). Collectively these results argue that fingolimod use, though alters tissue distribution of ILC3s, does not reduce antimicrobial peptide or Th17-associated cytokine production in the gut or skin.

### S1PR1 Ligands Have Immunomodulatory Effects on ILC1 and ILC3

**Figure 4:**
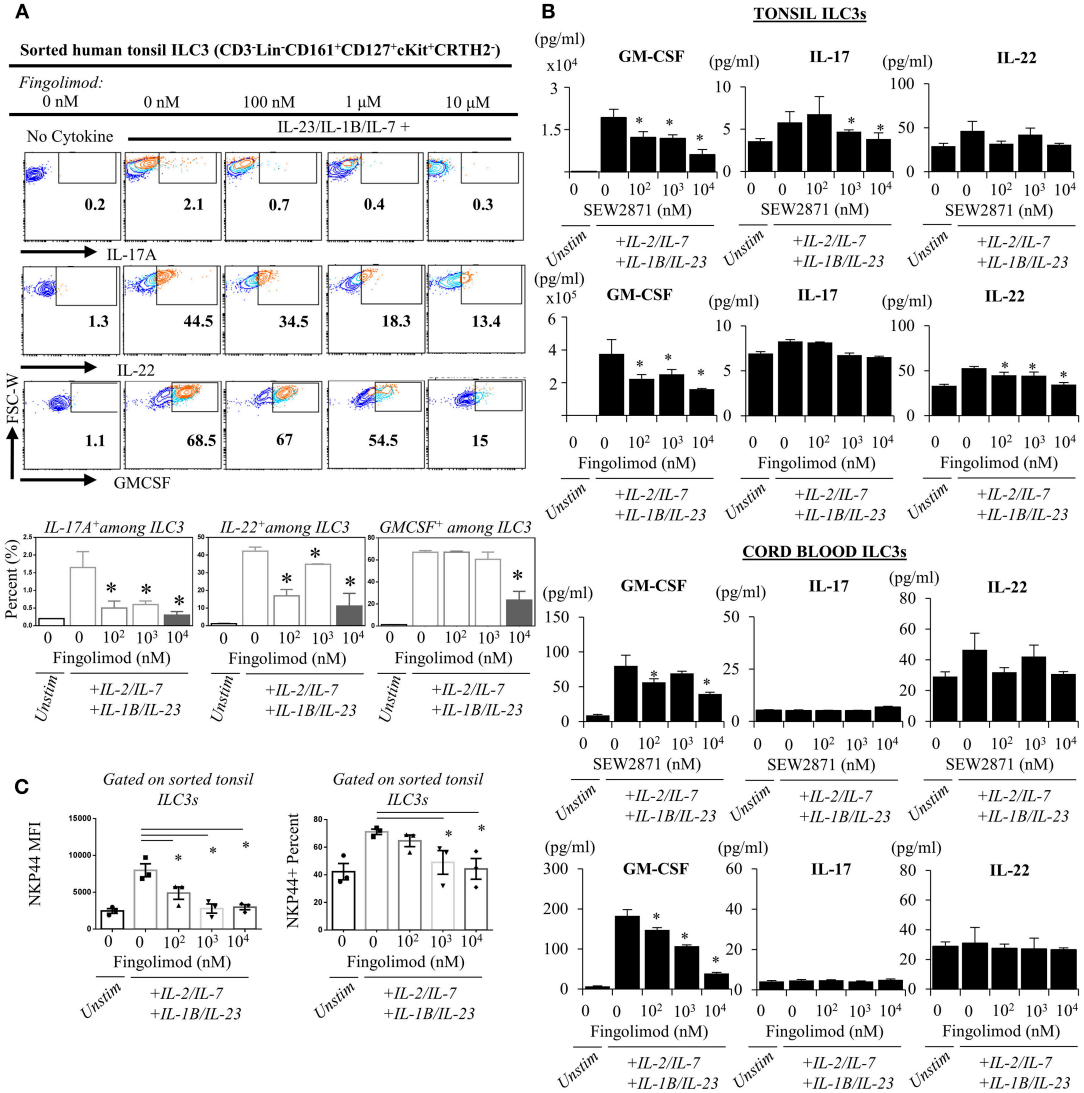
*Ex vivo* exposure of human ILC3s to S1P analogs inhibits production of ILC3-associated cytokines. **(A)** Sorted ILC3s from tonsils (gated as Lin-CD3-CD161+CD127+ckit+ CRTH2-) were cultured in complete medium at increasing doses of FTY720 and activated with IL-2, IL-23, IL-7, and IL-1B (20 ng/ml each) for 2–3 days, and PMA (50 ng/ml) /Inonomycin (1 μg/ml) stimulated for 4 h at 37°C for intracellular staining of indicated cytokines, representative flow plots (left) and quantified bar graphs for percentages of cells producing the indicated cytokines (right). **(B)** ELISA was performed from supernatants of “A” and SEW2871 exposed tonsil-derived (top panel) cord blood-derived (bottom panel) ILC3 cultures for ILC3-asssociated cytokines cord-derived ILC3s. **(C)** NKP44 surface expression by ILC3s were examined via flow cytometry, percent and mean fluorescence intensity (MFI) was quantified after culture with FTY720 for 3 days. *Indicates *p* < 0.05. The experiments were performed with triplicates and repeated for 3 times.

**Figure 5:**
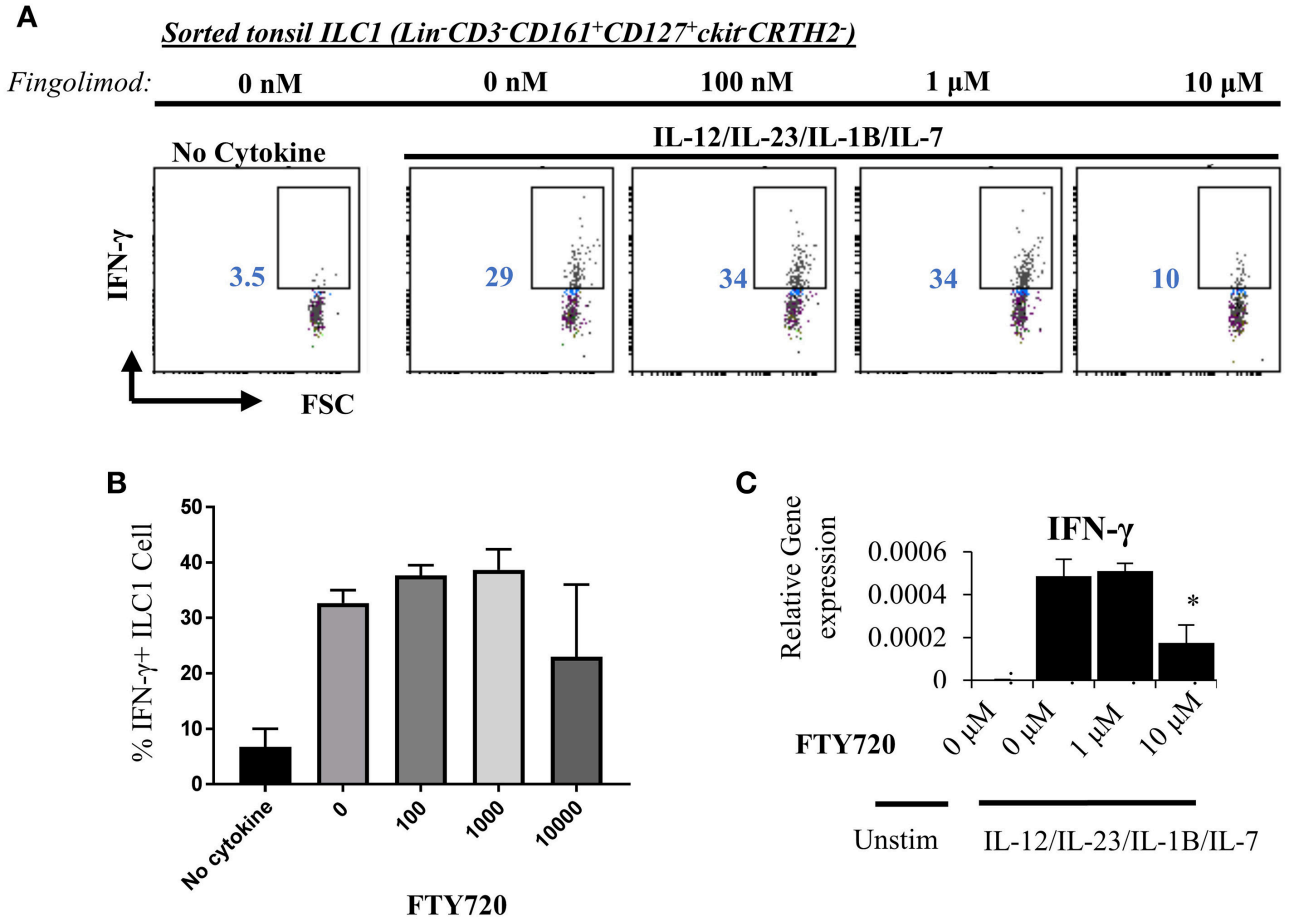
Fingolimod does not alter IFN-γ production by ILC1 at low doses. **(A)** ILC1s sorted from tonsils (gated as Lin-CD3-CD161+CD127+ckit-CRTH2-) were cultured in charcoal stripped FBS-supplemented media containing IL-12/IL-23/IL-1B/IL-7, 20 ng each, with increasing concentrations of FTY70 for 3 days IFN-γ production was measured with intracellular staining (**A**, a representative plot) and quantified **(B)** and mRNA levels were quantified via real time qPCR **(C)**.

Next, we explored the egress-independent impact of S1P analogs on human ILC3 and ILC1. We first cultured ILC3s and ILC1s sorted from healthy human tonsils or cord blood in charcoal-treated-FBS supplemented media (which is stripped of endogenous S1P) with different concentrations of fingolimod or SEW2871 for 3 days. ILC3 cultures are activated with a cytokine cocktail of IL-2/IL-7/IL-1B/IL-23 (50 ng/ml each). We did not observe a toxic effect of the drugs on ILC3 (Figure S5) or ILC1s (not shown) at doses below 10 μM when assessed by staining of apoptosis markers ANNEXINV and 7-AAD. Tonsil ILC3s produced GM-CSF, IL-22 and IL-17, when stimulated with IL-2/IL-7/IL-1B/IL-23 cytokine cocktail, and S1P analogs inhibited secretion and gene expression of these cytokines in a dose dependent manner (Figures 4A,B). As previously reported cord blood ILC3 subsets did not produce conventional ILC3 cytokines IL-22 or IL-17A (47). They produced GM-CSF in large quantities and S1P analogs also inhibited GMCSF production in a dose dependent manner (Figure 4B). We also checked the surface NKP44 expression by ILC3s cultured in the presence of S1P FTY720 (Figure 4C). IL-2/IL-7/IL-1B/IL-23 cytokine cocktail stimulation upregulated NKP44 surface expression by human tonsil ILC3s. Fingolimod exposure downregulated its surface expression in a dose-dependent manner (Figure 4C). Unlike ILC3s, fingolimod and SEW2871 inhibited IFN-γ production by ILC1 only at very high concentrations (10 μM) (Figures 5A–C). These *ex-vivo* treatment data contrast with *in vivo* fingolimod exposure data and suggest that, the observed increase in Th17-associated cytokines in the fingolimod injected mice gut *in vivo*, may come from sources other than ILC3s.

## Discussion

This study provides novel information regarding the regulation of ILCs by S1P analogs in humans primarily, and in mice. We show that all ILC subsets in humans express S1PR1 at the mRNA message level and specifically ILC1 and ILC3 at the protein level and migrate toward S1PR1 analogs *ex vivo*. Our data show that fingolimod, a currently used S1P analog, can sequester ILCs in secondary lymphoid organs in humans and mice, thus number of circulating blood ILCs as well as gut resident ILC3s drop drastically. This drop in the intestinal ILC3s, however, does not result in impaired production of mucosal cytokines, or anti-microbial peptides. Lastly, we provide evidence as to immunomodulatory effects of fingolimod on human ILC3s and ILC1s.

To the best of our knowledge, there is only a single recent report in which a murine ILC subset, ILC2s, was studied in relation to S1P/fingolimod (52). Interestingly, in that study, fingolimod injections were reported to block ILC2 migration from intestines to lung or bone marrow during an inflammatory state, thus increasing their abundance in the intestinal tissue. ILC3s have not been studied in that report (52). Moreover, there is no available research as to how and if S1P or its synthetic analogs regulate human non-NK ILC subsets. ILC egress from secondary lymphoid organs have not been investigated to this day. First studies by Gasteiger et al. in mice suggested that both lymphoid and non-lymphoid tissue residents ILCs are sedentary and are mostly maintained by local self-renewal during steady state (54). Using parabiotic mice, they also showed that even during two distinct forms of inflammatory state, tissue resident ILCs are of mostly host derived (54). More recently, Lim et al. showed that, the circulating blood ILC3 population in humans contained a number of ILC precursors which can migrate into tissues, and *in situ*, differentiate into all three subsets of ILCs (47). This study employed a combination of *in vivo* adoptive transfer as well as serial dilution and cloning studies *ex vivo* (47). Our data on human ILCs in fingolimod-treated MS patients, combined with fingolimod-injected mice, show that ILC circulation in both species could be interrupted by fingolimod. The increase in the LNs and the decrease in a non-lymphoid tissue, small intestine, show that egress from secondary lymphoid tissue to lymph is regulated by S1P receptors. Given that FTY720 binds four of the S1P receptors, and ILCs can migrate toward SEW2871, a selective S1PR1 agonist, and SEW2871 pre-treatment blocks ILC1/ILC3 migration as efficiently as FTY720 pre-treatment, we propose that S1PR1 may be the major receptor for migration of human ILC1 and ILC3 toward S1P.

Second important piece of information our present study reveals is that long term fingolimod use does not compromise barrier immunity-associated cytokines, or antimicrobial peptides. Several studies showed that ILC3s and Th17 cells are crucial for homeostasis in the gut (28, 31). ILC3s are critical in various processes in the gut including tolerance induction (55), production of antimicrobial proteins (56), epithelial regeneration (53, 57), in addition to their role in protective immunity against fungal or extracellular pathogens. Whether the number of ILC3s in the small intestine changes over extended period of fingolimod use, and consequently impact those processes is a legitimate concern. Our results are interesting in that, although intestinal lamina propria resident ILC3s drop in numbers, probably due to entrapment of circulating ILCs in the LNs, ILC3-signature cytokines and antimicrobial peptides remained significantly high after prolonged fingolimod injections.

Lastly our data provide evidence for immunomodulatory effects of S1P analogs on human ILCs. S1P was previously shown to inhibit IFN-γ and IL-4 production by CD4+ T cells (58). Others also reported that FTY720 was able to inhibit IL-33/IL12-induced IFN-γ production by CD8+ T cells (59). Our data reveal that SEW2871, a selective S1PR1 agonist, and FTY720, a non-selective S1PR1 agonist can both block GM-CSF, IL-17A, and IL-22 production by ILC3s in a dose-dependent manner *ex vivo*. However, intraperitoneal injection or oral administration of fingolimod did not result in the reduction of these cytokines *in vivo* in the intestinal tissue, despite a drop in ILC3 numbers in the small intestine lamina propria. This suggests that sources other than ILC3s such as Th17, γδ T, mucosal-associated invariant T (MAIT) cells or neutrophils may contribute to the production of these cytokines. It appears that long term fingolimod injection may have activated remaining sources of such cytokines in the gut, as a result, more IL-22, GM-CSF and IL-17A is produced compared with vehicle treated mice intestinal tissue cultures.

In summary, this work reveals that human and murine ILCs utilize S1P receptors for egress from secondary lymphoid organs, and that ILCs could be targeted by S1P analogs in both humans and mice.

## Ethics Statement

All protocols were approved by the local ethics committee for animal research (#2017/458). All the experiments were performed in accordance with the relevant guidelines and regulations established by Erciyes University Institutional Review Board in accordance with Helsinki Guidelines.

## Author's Note

Part of the results in this study were presented as poster and oral presentation in the AAI 2018 meeting in Texas, USA, and WIRM Meeting in 2017 in Davos, Switzerland, respectively.

## Author Contributions

AE, AV, MY, HD, HC, MM, MO, and MK designed the study, reviewed the manuscript interpreted the results. FO, SE, and ZA, performed cell sorting, and most experiments. YH, OK, ET, and IK performed chemotaxis experiments and reviewed the manuscript. MC helped with oral fingolimod treatment experiments in mice. MK provided cord blood samples. AV and IK organized tonsil samples. AE wrote manuscript and supervised the study.

### Conflict of Interest Statement

The authors declare that the research was conducted in the absence of any commercial or financial relationships that could be construed as a potential conflict of interest.

## Supplementary Material

Figure S1 Expression levels of S1P receptors by murine and human ILC subsets. **(A)** S1P receptor expression by murine intestinal ILC3 and Th17 RNASeq data from GSE70596 (left). S1P receptor expression by human ILC3 and NK cells, microarray data obtained from GSE63197 (right). **(B)** Relative gene expression of S1P receptors by murine intestinal ILC2, ILC3, and T cells measured by real time qPCR. **(C)** Sorted human ILC1s are pretreated with either serum free media alone or with pertussis toxin for 2 h, then cell migration toward 1 μM FTY20 was quantified using trans-well migration assay. Percentage of cells migrating to bottom chamber was shown. **(D)** Sorted human total ILCs cells were pretreated with either serum free media, or FTY720, or SEW2871 for 2 h, then cell migration toward FBS was quantified using trans-well migration assay. No-FBS condition measures spontaneous migration toward serum free media. **(E)** Sorted human Tonsil ILC1 (CD3-Lin-CD161+CD127+cKit-CRTH2-), ILC3 (CD3-Lin-CD161+CD127+cKit+CRTH2-) and T cells (CD3+Lin-CD161-) were stained with S1PR1 or isotype antibody. ^*^ indicates *p* value < 0.05.

Figure S2 Gating strategy for PBMCs obtained from humans and mice. **(A)** A representative sequential gating for human peripheral blood ILC subsets. Top panel shows untreated MS patient blood PBMCs, bottom panel shows fingolimod receiving-patient PBMCs. **(B)** A representative sequential gating of mouse peripheral blood ILC3s for a blood sample obtained from IL-23RGFP reporter mouse.

Figure S3 Murine ILC gating Strategy. **(A)** Gating of mouse ILCs using Gata3 and Rorγt staining in blood, spleen, small intestine (SI) inguinal lymph node (LN). **(B,C)** Gating of ILC3s in the small intestine (SI) using IL-23RGFP reporter mice. A representative flow plot for one mouse.

Figure S4 Oral fingolimod administration decreases murine small intestine lamina propria ILC3 numbers in mice but does not reduce antimicrobial peptide production. **(A)** Representative flow plots for Gata3+ ILC2 distribution in the organs of fingolimod- or vehicle fed mice for 30 days. **(B)** Absolute number of CD3+ T and B220+ B or CD45+ total lymphocytes in the blood, mesenteric lymph node (LN) and small intestine of fingolimod- or vehicle fed mice for 30 days. **(C)** Absolute number of total lymphocytes, CD45^medium^CD90.2^high^ ILC3s in the small intestine or colon lamina propria of anti-CD40 injected mice, day 2 of injection. Five mice per group were used. Experiment was repeated 2 times. **(D)** 1 cm piece of ileum or colon from mice treated orally with fingolimod or vehicle for 15 days was examined for gene expression of indicated antimicrobial peptides and cytokines via real-time qPCR. **(E)** 1 cm^2^ piece of skin from mice treated orally with fingolimod or vehicle for 15 days was examined for gene expression of indicated antimicrobial peptides and cytokines via real-time qPCR. Five mice per group were used. Skins were pooled and run as technical triplicates. **(F)** Small intestine lamina propria lymphocytes were isolated from 30-day fingolimod treated mice, B220 vs. CD45 or FSC vs. CD45 flow plots were shown for one mouse per group. ^*^Indicates *p* < 0.05.

Figure S5 Fingolimod does not have toxic effects on human ILC3 below 10 μM doses. A representative flow plot for 7AAD and ANNEXIN V staining of sorted ILC3 (CD3^−^Lin^−^CD161^+^CD127^+^cKit^+^CRTH2^−^) cultured in the presence of absence of activating cytokines for 3 days at varying fingolimod doses (Top panel). The percentages of early apoptotic (ANNEXINV^+^7AAD^−^), late apoptotic (ANNEXINV^+^7AAD^+^) and live (ANNEXINV^−^7AAD^−^) cells quantified.

Figure S6 Primer list.

Table S1 MS Patient age and sex information.
