# Supplementary material for: Fingolimod Alters Tissue Distribution and Cytokine Production of Human and Murine Innate Lymphoid Cells
Source: Front Immunol. 2019 Feb 13;10:217. doi: 10.3389/fimmu.2019.00217 (PMC6385997; doi:10.3389/fimmu.2019.00217)
Supplement: Figure S1 — Expression levels of S1P receptors by murine and human ILC subsets. (A) S1P receptor expression by murine intestinal ILC3 and Th17 RNASeq data from GSE70596 (left). S1P receptor expression by human ILC3 and NK cells, microarray data obtained from GSE63197 (right). (B) Relative gene expression of S1P receptors by murine intestinal ILC2, ILC3, and T cells measured by real time qPCR. (C) Sorted human ILC1s are pretreated with either serum free media alone or with pertussis toxin for 2 h, then cell migration toward 1 μM FTY20 was quantified using trans-well migration assay. Percentage of cells migrating to bottom chamber was shown. (D) Sorted human total ILCs cells were pretreated with either serum free media, or FTY720, or SEW2871 for 2 h, then cell migration toward FBS was quantified using trans-well migration assay. No-FBS condition measures spontaneous migration toward serum free media. (E) Sorted human Tonsil ILC1 (CD3-Lin-CD161+CD127+cKit-CRTH2-), ILC3 (CD3-Lin-CD161+CD127+cKit+CRTH2-) and T cells (CD3+Lin-CD161-) were stained with S1PR1 or isotype antibody. * indicates p value < 0.05. [file Data_Sheet_1.pdf]

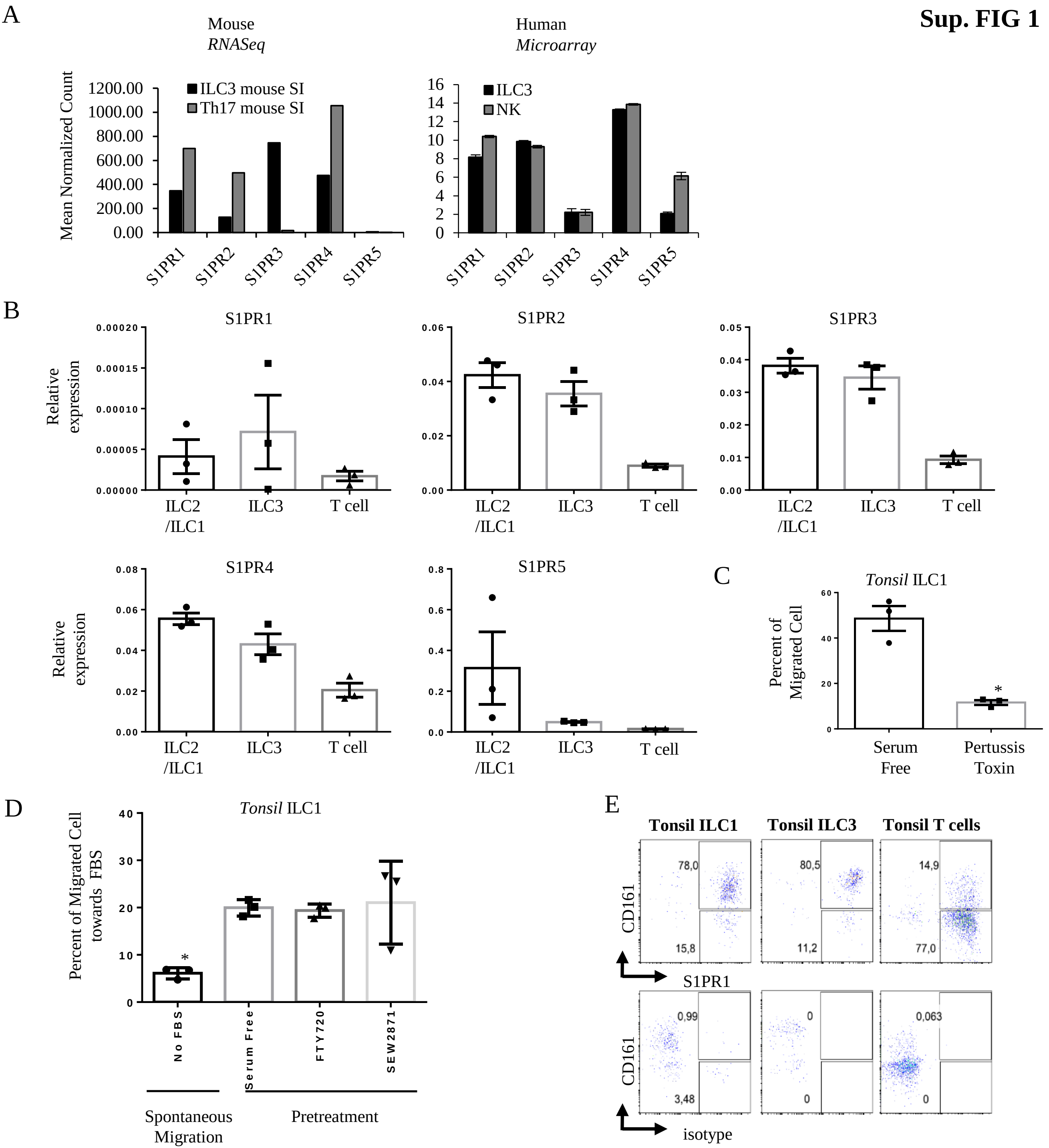
Sup. FIG 1
A
Mouse
RNASeq
Human
Microarray
Mean Normalized Count
ILC3 mouse SI
Th17 mouse SI
ILC3
NK
S1PR1
S1PR2
S1PR3
S1PR4
S1PR5
B
Relative expression
ILC2 /ILC1
ILC3
T cell
C
Tonsil ILC1
Percent of Migrated Cell
Serum Free
Pertussis Toxin
D
Percent of Migrated Cell towards FBS
No FBS
Serum Free
FTY720
SEW2871
Spontaneous Migration
Pretreatment
E
Tonsil ILC1
Tonsil ILC3
Tonsil T cells
CD161
S1PR1
isotype
78,0
15,8
80,5
11,2
14,9
77,0
0,99
3,48
0
0
0,063
0

A

Sup. FIG 2

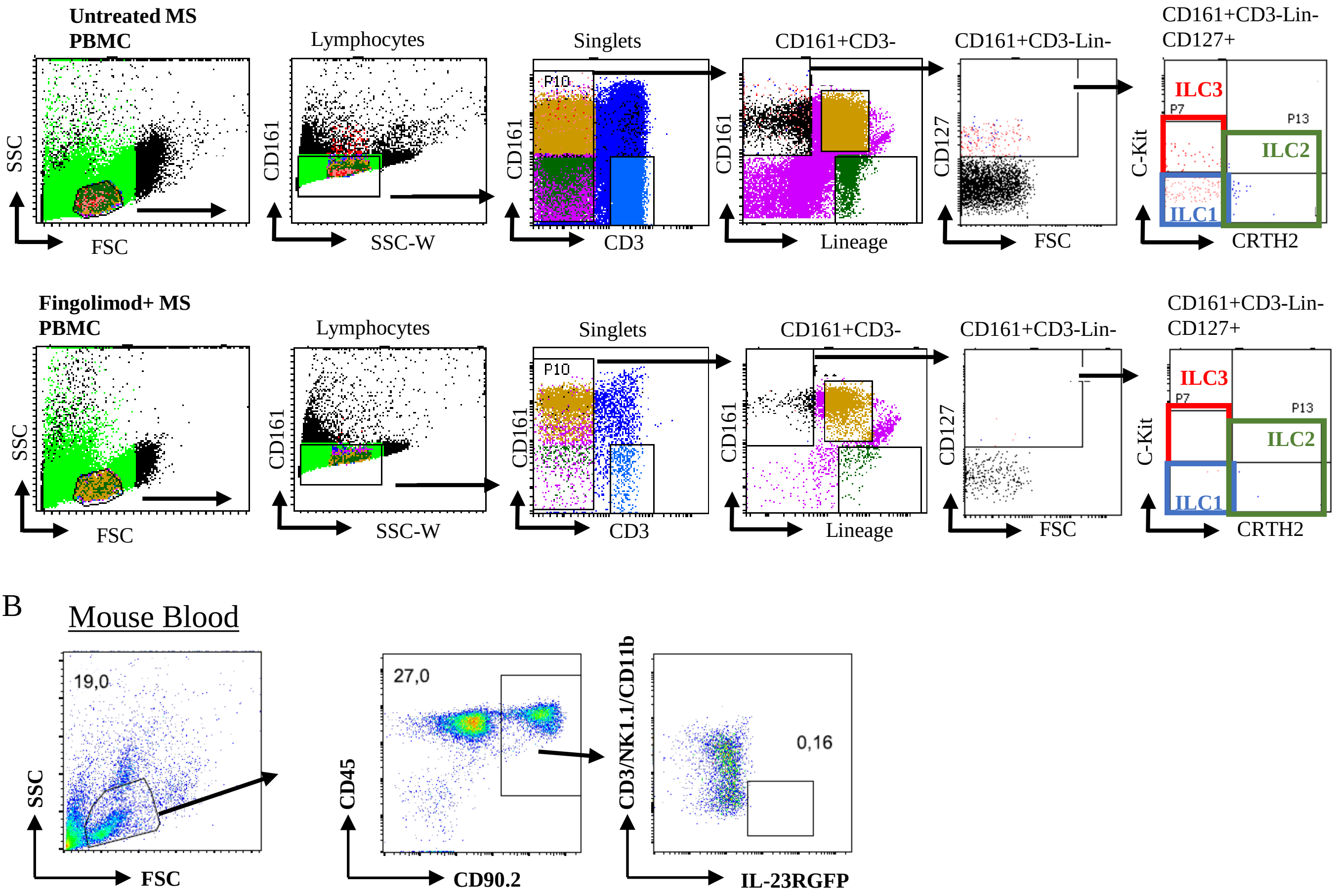

Untreated MS PBMC
SSC
FSC
Lymphocytes
CD161
SSC-W
Singlets
P10
CD3
CD161+CD3-
Lineage
CD161+CD3-Lin-
CD127
CD161+CD3-Lin- CD127+
ILC3
P7
P13
ILC2
ILC1
C-Kit
CRTH2
Fingolimod+ MS PBMC
B
Mouse Blood
19,0
27,0
CD45
CD90.2
CD3/NK1.1/CD11b
0,16
IL-23RGFP

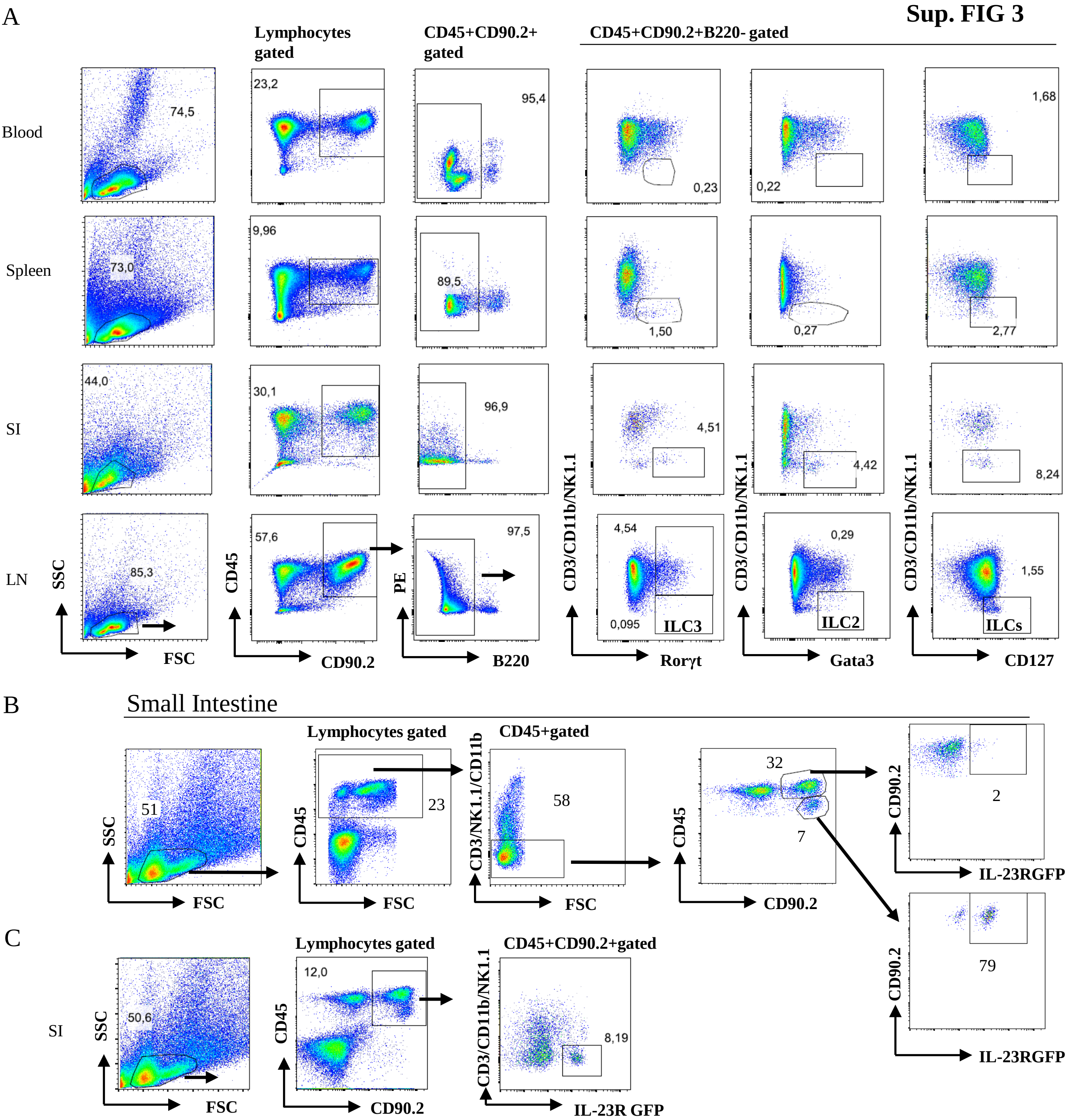

Sup. FIG 3
A
Lymphocytes gated
CD45+CD90.2+ gated
CD45+CD90.2+B220- gated
Blood
74,5
23,2
95,4
0,23
0,22
1,68
Spleen
73,0
9,96
89,5
1,50
0,27
2,77
SI
44,0
30,1
96,9
4,51
4,42
8,24
LN
85,3
57,6
97,5
4,54
0,095
ILC3
0,29
ILC2
1,55
ILCs
SSC
FSC
CD45
CD90.2
PE
B220
CD3/CD11b/NK1.1
Rorγt
Gata3
CD127
B
Small Intestine
Lymphocytes gated
CD45+gated
51
23
58
32
7
2
79
CD3/NK1.1/CD11b
IL-23RGFP
C
SI
Lymphocytes gated
CD45+CD90.2+gated
50,6
12,0
8,19
IL-23R GFP

Sup. FIG 4

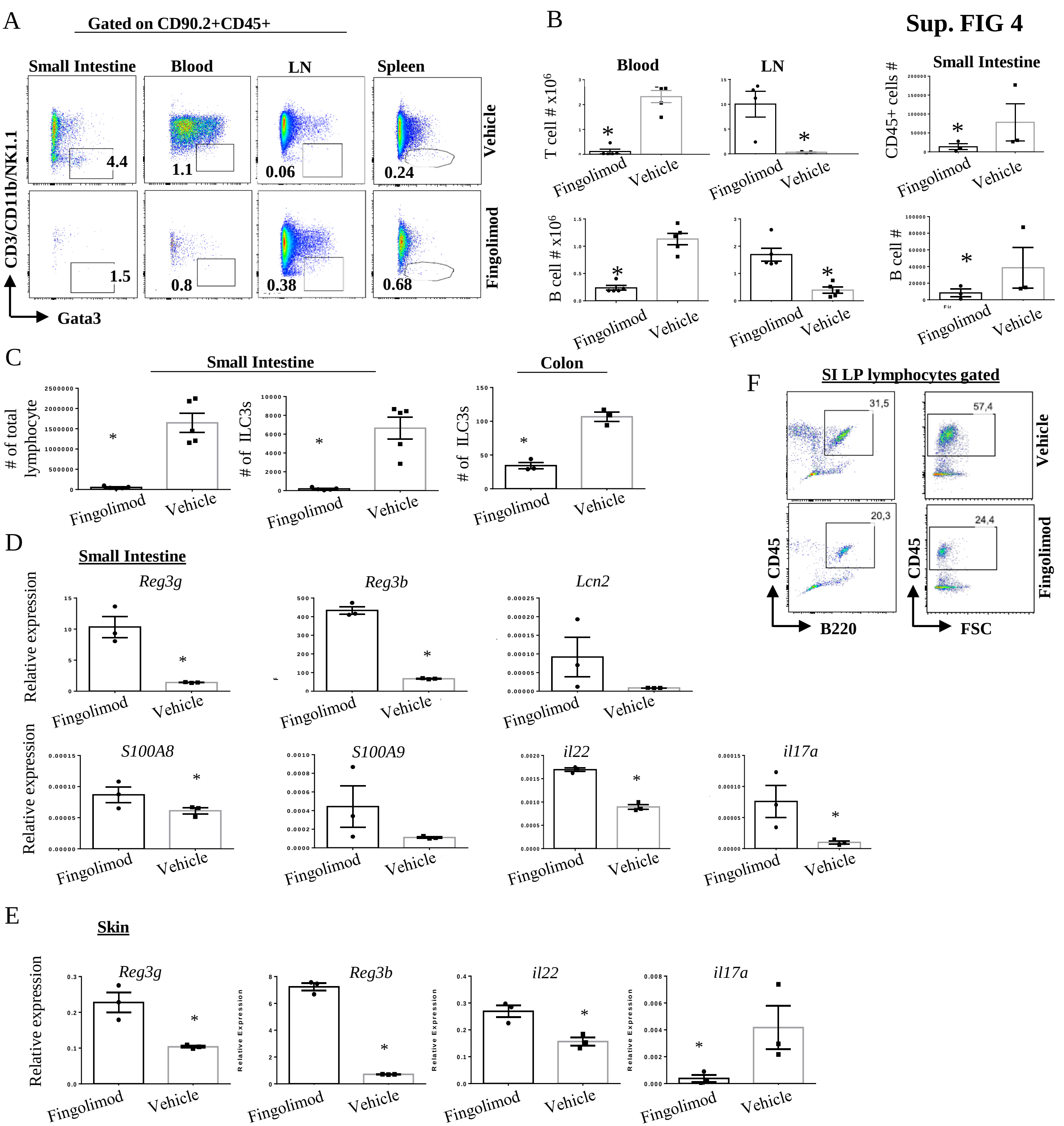
A
Gated on CD90.2+CD45+
Small Intestine
Blood
LN
Spleen
Vehicle
Fingolimod
4.4
1.1
0.06
0.24
1.5
0.8
0.38
0.68
CD3/CD11b/NK1.1
Gata3
B
Blood
LN
Small Intestine
T cell # x10^6
CD45+ cells #
B cell # x10^6
B cell #
Fingolimod
Vehicle
C
Small Intestine
Colon
# of total lymphocyte
# of ILC3s
D
Small Intestine
Reg3g
Reg3b
Lcn2
S100A8
S100A9
il22
il17a
Relative expression
E
Skin
Relative Expression
F
SI LP lymphocytes gated
31,5
57,4
20,3
24,4
CD45
B220
FSC

Sup. FIG 5

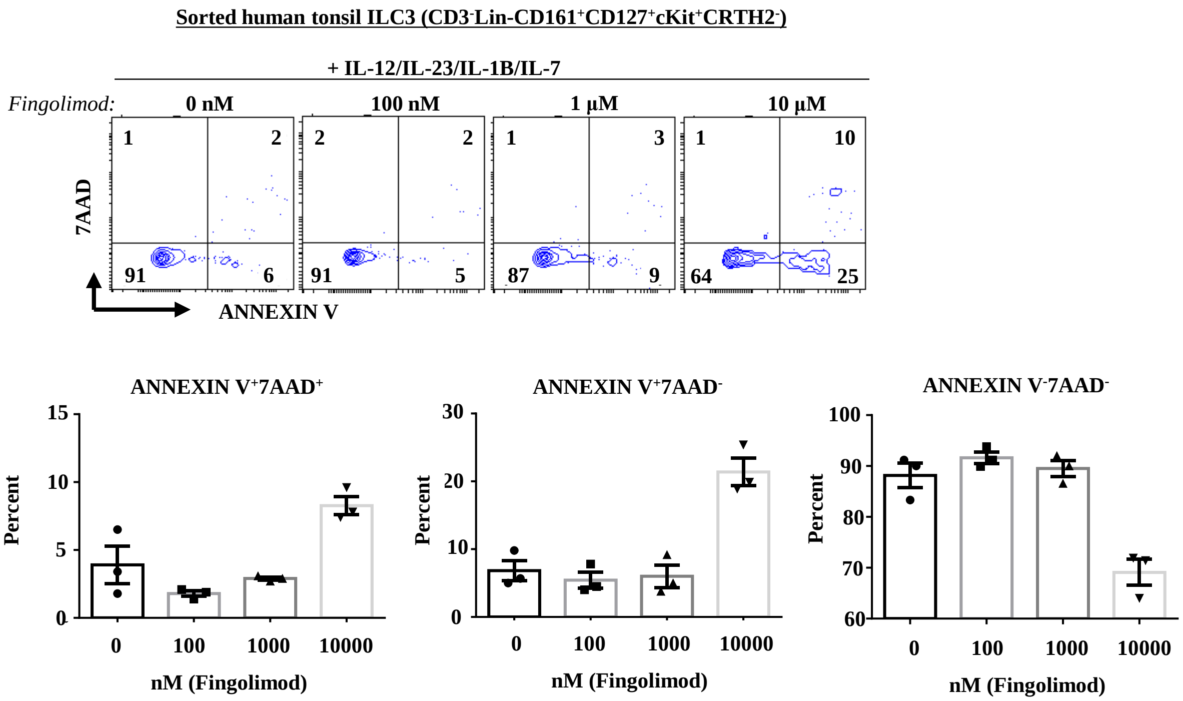

Sorted human tonsil ILC3 (CD3-Lin-CD161+CD127+cKit+CRTH2-)
+ IL-12/IL-23/IL-1B/IL-7
Fingolimod:
0 nM
100 nM
1 µM
10 µM
7AAD
ANNEXIN V
1
2
91
6
2
2
91
5
1
3
87
9
1
10
64
25
ANNEXIN V+7AAD+
ANNEXIN V+7AAD-
ANNEXIN V-7AAD-
Percent
0
5
10
15
20
30
60
70
80
90
100
0
100
1000
10000
nM (Fingolimod)

**Gene Name**	**Primer Sequence**	**Reference**
hS1PR1_FW	GCTCTCCGAACGCAACTTC	[1]
hS1PR1_RV	GGTGGTTCGATGAGTGATCC	
hS1PR2_FW	CAAGTTCCACTCGGCAATGT	[2]
hS1PR2_RV	CAGGAGGCTGAAGACAGAGG	
hS1PR3_FW	TCAGGGAGGGCAGTATGTTC	[2]
hS1PR3_RV	GAGTAGAGGGGCAGGATGGT	
hS1PR4_FW	GTCTTTGGCTCCAACCTCTG	[3]
hS1PR4_RV	CGGAAGGAGTAGATGATGGG	
hS1PR5_FW	GGCGCGCACCTGTCCTGTAC	
hS1PR5_RV	TCGGGTCTCTGCCGCAGGAG	
mS1PR1_FW	GGAGGTTAAAGCTCTCCGC	
mS1PR1_RV	CGCCCCGATCTTCAAC	
mS1PR2_FW	GCGTGGTCACCATCTTCTCC	[4]
mS1PR2_RV	CGTCTGAGGACCAGCAACATC	
mS1PR3_FW	CATCGCCTTCCTCATCAGTATCTTC	[4]
mS1PR3_RV	CACAATCACTACGGTCCGCA	
mS1PR4_FW	CCACAGCCTCCTCATTGTC	[4]
mS1PR4_RV	TCAGCATCCCTAGCCCTC	
mS1PR5_FW	ACTGCTTAGGACGCCTGGAA	[4]
mS1PR5_RV	CCGCACCTGACAGTAAATCCTT	
mIFNg_FW	TCAAGTGGCATAGATGTGGAAGAA	
mIFNg_RV	TGGCTCTGCAGGATTTTCATG	
mLcn2_FW	GGCCCTGAGTGTCATGTGTC	
mLcn2_RV	TTCTGATCCAGTAGCGACAGC	
mReg3g_FW	CCGTGCCTATGGCTCCTATTG	
mReg3g_RV	GCACAGACACAAGATGTCCTG	
mReg3B_FW	TACTGCCTTAGACCGTGCTTTCTG	
mReg3B_RV	GACATAGGGCAACTTCACCTCACA	
mS100a8_FW	TGCGATGGTGATAAAAGTGG	
mS100a8_RV	GGCCAGAAGCTCTGCTACTC	
mS100a9_FW	CACAGTTGGCAACCTTTATG	
mS100a9_RV	CAGCTGATTGTCCTGGTTTG	

Sup. Table 1

	Fingolimod+ (MS Patient)		***Untreated (MS Patient)***	
	Male	Female	Male	Female
Patients (n)	4	10	4	7
Mean Age	46.5	41.33	30.25	32.83

Supplementary References

1.Sic, H., Kraus, H., Madl, J., Flittner, K.-A., Lilly Von M€ Unchow, A., Pieper, K., … Eibel, H. (2014). Sphingosine-1-phosphate receptors control B-cell migration through signaling components associated with primary immunodeficiencies, chronic lymphocytic leukemia, and multiple sclerosis. *Journal of Allergy and Clinical Immunology*, *134*, 420–428.e15. https://doi.org/10.1016/j.jaci.2014.01.037

2.Gu, Y., Forostyan, T., Sabbadini, R., & Rosenblatt, J. (2011). Epithelial cell extrusion requires the sphingosine-1-phosphate receptor 2 pathway. *Journal of Cell Biology*, *193*(4), 667–676. https://doi.org/10.1083/jcb.201010075

3.He, X., H'ng, S.-C., Leong, D. T., Hutmacher, D. W., & Melendez, A. J. (2010). Sphingosine-1-Phosphate Mediates Proliferation Maintaining the Multipotency of Human Adult Bone Marrow and Adipose Tissue-derived Stem Cells. *Journal of Molecular Cell Biology*, *2*(4), 199–208. https://doi.org/10.1093/jmcb/mjq011

4.Orr Gandy, K., Canals, D., Adada, M., Wada, M., Roddy, P., Snider, A., … Obeid, L. (2012). Sphingosine 1-Phosphate induces filopodia formation through S1P2R activation of ERM proteins. *Biochemical Journal*, *672*, 661–672. https://doi.org/10.1042/BJ20120213
