# Supplementary material for: Fingolimod Alters Tissue Distribution and Cytokine Production of Human and Murine Innate Lymphoid Cells
Source: Front Immunol. 2019 Feb 13;10:217. doi: 10.3389/fimmu.2019.00217 (PMC6385997; doi:10.3389/fimmu.2019.00217)
Supplement: Supplementary file 2 [file Data_Sheet_2.docx]

**Supplementary References**

1.Sic, H., Kraus, H., Madl, J., Flittner, K.-A., Lilly Von M€ Unchow, A., Pieper, K., … Eibel, H. (2014). Sphingosine-1-phosphate receptors control B-cell migration through signaling components associated with primary immunodeficiencies, chronic lymphocytic leukemia, and multiple sclerosis. *Journal of Allergy and Clinical Immunology*, *134*, 420–428.e15. https://doi.org/10.1016/j.jaci.2014.01.037

2.Gu, Y., Forostyan, T., Sabbadini, R., & Rosenblatt, J. (2011). Epithelial cell extrusion requires the sphingosine-1-phosphate receptor 2 pathway. *Journal of Cell Biology*, *193*(4), 667–676. https://doi.org/10.1083/jcb.201010075

3.He, X., H’ng, S.-C., Leong, D. T., Hutmacher, D. W., & Melendez, A. J. (2010). Sphingosine-1-Phosphate Mediates Proliferation Maintaining the Multipotency of Human Adult Bone Marrow and Adipose Tissue-derived Stem Cells. *Journal of Molecular Cell Biology*, *2*(4), 199–208. https://doi.org/10.1093/jmcb/mjq011

4.Orr Gandy, K., Canals, D., Adada, M., Wada, M., Roddy, P., Snider, A., … Obeid, L. (2012). Sphingosine 1-Phosphate induces filopodia formation through S1P2R activation of ERM proteins. *Biochemical Journal*, *672*, 661–672. https://doi.org/10.1042/BJ20120213
